# Nutritional and Bioactive Components of Pomegranate Waste Used in Food and Cosmetic Applications: A Review

**DOI:** 10.3390/foods10030657

**Published:** 2021-03-19

**Authors:** Katie Ko, Younas Dadmohammadi, Alireza Abbaspourrad

**Affiliations:** Department of Food Science, College of Agriculture and Life Sciences, Cornell University, Ithaca, NY 14853, USA; khk48@cornell.edu (K.K.); younas@cornell.edu (Y.D.)

**Keywords:** pomegranate, waste, food packaging, food additive, skin health, cosmetics

## Abstract

Pomegranate (*Punica granatum* L.) is a fruit that is rich in bioactive compounds that has a biowaste (rind and seed) with the potential to be converted into value-added products in a wide variety of applications. Recent studies have demonstrated the potent antioxidant and antimicrobial effects of using pomegranate rind and seed as natural food additives, thus making researchers incorporate them into bioplastics and edible coatings for food packaging. Additionally, these components have shown great plasticizing effects on packaging materials while extending the shelf life of food through active packaging. Even within skin health applications, pomegranate seed oil and its bioactive compounds have been particularly effective in combating UV-induced stresses on animal skin and in-vitro models, where cells and microorganisms are separated from the whole organism. They have also aided in healing wounds and have shown major anti-inflammatory, analgesic, and anti-bacterial properties. This review highlights all of the relevant and recent food and skin health applications found in the value-added conversion of pomegranate biowaste. The lack of research in particular areas and future outlook are also discussed.

## 1. Introduction

Annually, the United States disposes of roughly 133 billion pounds (31%) of its food supply [1]. Of this, approximately 61.2 billion pounds of food waste end up in landfills [2]. This excessive waste is not only detrimental to the environment, but it also contributes significantly to climate change issues, as food waste produces 18% of the total methane emissions in U.S. landfills. Contributing further to food waste and methane emissions are the 30% to 40% of fruits and vegetables that end up in waste [3]. Among these fruits and vegetables is pomegranate, the majority of which is considered to be waste after extracting juice.

Pomegranate’s rind and seeds, which account for roughly 54% of the fruit, are the waste components of pomegranate after juice extraction. California will be used as a model to showcase pomegranate production in the United States, as California produces over 90% of the pomegranates grown domestically, with the majority of the production based in the San Joaquin Valley [4,5]. In 2018 alone, California produced approximately 218,000 tons of pomegranates, making roughly 118,000 tons of pomegranate rind and seed waste [6]. This problem is compounded when the resources and energy that are used in producing the wasted food are taken into consideration [2]. To produce 5.6 tons of pomegranates per acre in San Joaquin Valley, 41 gallons of fuel and over 1.2 million gallons of water are consumed [7]. On a global scale, there are three-million tons of total pomegranate production, resulting in approximately 1.62 million tons of waste [8]. For comparison measures, 68 million tons of oranges are produced globally each year, which is 8.5% of total fruit production, and it results in 15 to 25 million tons of orange biowaste [9]. The sheer amount of waste that is produced for each edible percentage of pomegranate makes it important to look for proper methods of optimizing the nutritional and bioactive components of pomegranate waste and then convert this waste into value-added products to save energy, sustain resources, and protect the environment. Even if the extraction of nutritional and valuable components of pomegranate may not be feasible due to cost, looking into these applications is an important step to take in the future direction for a sustainable way to use this excessive waste.

### 1.1. Properties and Chemical Composition of Pomegranate

**Figure 1:**
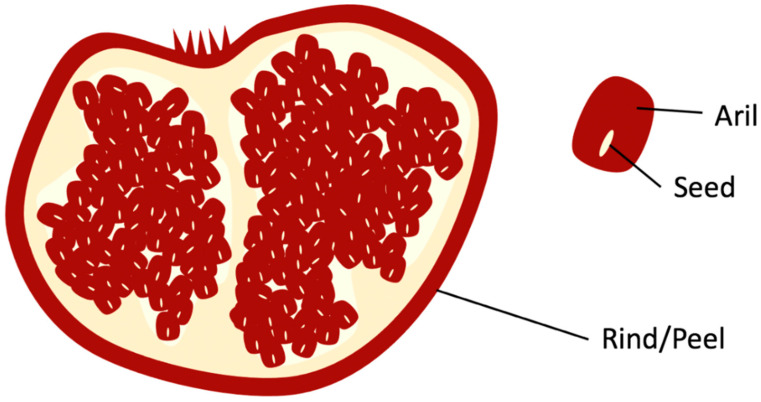
Rind and Seed of Pomegranate.

Pomegranate’s arils are depicted in Figure 1 as the edible red pulp that surrounds the seed that is used by juice manufacturing companies to produce pomegranate juice. The remaining solid waste from pomegranates, after juice extraction, rind, and seed (Figure 1), contains various bioactive and nutritional components, such as flavonoids (e.g., anthocyanins), hydrolyzable tannins (e.g., punicalagin and ellagic acid), and fatty acids (e.g., punicic acid). These components in pomegranate biowaste have various potential value addition applications in food and skin health [10].

**Figure 2:**
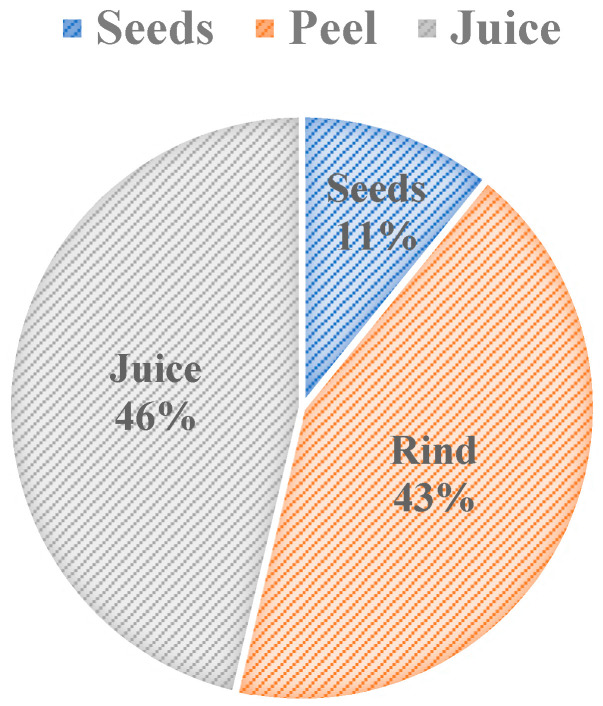
Weight Percent Composition of Pomegranate [10,13,14].

Figure 2 shows the weight percent composition breakdown of pomegranate parts. The breakdown shows that 46% *w/w* of pomegranate is used as a juice, and the rest of it is considered to be waste. The pomegranate rind is one of the waste components that comprises 43% *w*/*w* of the fruit. Seeds are another waste component of pomegranate and compose 11% *w*/*w* of the fruit (Figure 2). The oil content that is extracted from pomegranate seeds varies in weight percentages, depending on their cultivars, and it constitutes approximately 7.6–20% *w*/*w* of the pomegranate seed [11,12]. The oil content of pomegranate varies depending on the climate of the growing region, the maturity of the fruit, cultivation practices, and storage conditions [12]. To take cultivar variation in weight composition—that is a result of using different cultivars—into account, the average composition from three sources was used in Figure 2. Pomegranate processing factories will benefit from utilizing the waste components because the majority of the pomegranate fruit is disposed of as waste.

### 1.2. Pomegranate Seed

**Figure 3:**
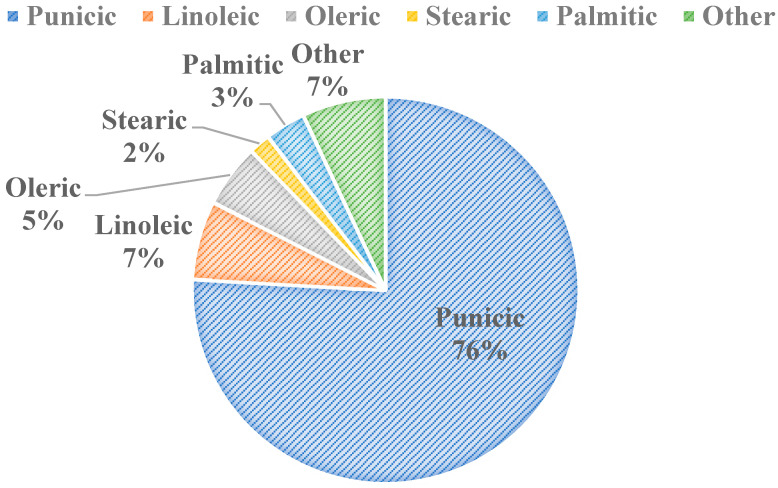
Major Fatty Acids in Pomegranate Seed Oil [11,20].

Figure 3 shows the five major fatty acid components found in pomegranate seed oil (PSO). There are 45 identified different fatty acids in PSO, with conjugated fatty acids making up over 80% *w*/*v* of its composition [14]. The 45 fatty acids were identified using gas chromatography coupled with mass spectroscopy [15]. Punicic acid is the main fatty acid in PSO, being followed closely by linoleic and oleic acids (Figure 3). Some studies show the health benefits of specific fatty acid components within the seed oil, such as punicic acid’s role in preventing diabetes and related obesities [16]. It has also been shown that punicic acid can inhibit skin cancer [17] and prevent type 2 diabetes [18] in rats, and it possesses anti-diabetic, anti-obesity, anti-inflammatory, anticancer, and antioxidant properties [19]. Similarly, a wide scope of applications for PSO extracts as a whole exist, including in food packaging, fat substitution, animal food production and functional ingredient, and antimicrobial agent and pharmaceutical capacities [14]. There is an increasing demand and acceptance for PSO for consumers in the cosmetics, food, and pharmaceutical industries globally due to PSO’s valuable phytochemical composition and functional properties. The oil also has feasible extraction procedures, and it has applications in cosmetic products, especially in Europe [14]. PSO’s applications in both the food and skin health sectors will be discussed in detail.

Table 1 shows some of the chemical properties of the rind and seed to help outline the various applications that add value to pomegranate’s biowaste as functional ingredients in food. For instance, pomegranate seed is a source of dietary fiber that has applications as a food additive in fiber-enriched products, including dough [21] and chicken nuggets [22].

Extractions. Traditional extraction methods for edible PSO include solvent extraction, distillation, cold pressing, hot pressing, etc., but these methods can impair the bioactive components of PSO that can be thermally degraded. Newer extraction methods include superficial fluid extraction (SFE), and enzyme, ultrasound, microwave, and pulsed electric field-assisted extractions [14]. PSO can be extracted while using different organic solvents, such as petroleum benzene and hexane, by ultrasonic and microwave-assisted extraction procedures. However, this extraction procedure does not provide any significant difference in the amount of phenolic content extracted from PSO using different solvents due to the polarity of phenolic components in the seed oil and the non-polarity of the organic solvents [23]. SFE is another extraction method that is efficient, selective, clean, and safe to the environment. Supercritical CO_2_ can be used as a solvent with modifiers, such as water, ethanol, and hexane, for the SFE method. The use of superficial CO_2_ results in more selective extractions for components in PSO than the ultrasonic and microwave-assisted extraction methods. The extraction yields were the highest using modifiers in the following order: hexane, ethanol, and then water [23]. Propane can be used as an alternative, relatively inexpensive solvent that does not leave toxic residues and requires less pressure for extraction to a magnitude of ten, in order to reduce the cost of extraction and environmental concerns using SFE with supercritical CO_2_. Furthermore, propane increased the extraction of the total amount of PSO from 13.06% *w*/*w* using supercritical CO_2_ to 17.12% *w*/*w* [24]. A downside of using the SFE method is the high capital equipment cost, although it provides more benefits than traditional extraction methods [14].

### 1.3. Pomegranate Rind

Pomegranate rind is the main non-edible portion that constitutes approximately 43% *w*/*w* of the fruit (Figure 2). It is a source of bioactive compounds, including flavonoids, complex polysaccharides, minerals, and hydrolyzable tannins, such as punicalagin, ellagic, and gallic acid (Table 2) [10]. Pomegranate rind has a variety of applications in wastewater treatment, including being used in the removal of phenolic compounds [25], in the removal of dye from wastewater by conversion of pomegranate rind to activated carbon [26], and as renewable energy material sources [27,28]. Pomegranate rind is a rich source of dietary fiber and pectin, as shown in Table 1. In one study, pomegranate rind powder was added to the diet of hypercholesteremic rats as a source of dietary fiber, and it has been shown to combat the risks that are associated with hypercholesterolemia such as lipid peroxidation [29]. Pomegranate rind has also been supplemented in foods, such as cookies, to enhance its nutritional benefits [30]. The rind’s phenolics assisted in improving oxidative stability during food storage in addition to significantly increasing the cookies’ dietary fiber content, which allows the product to be marketed towards health-conscious consumers. The phenolics in cookies also improve the antioxidant activity in the Gastro-intestinal Tract at a 7.5% *w*/*w* of rind extract and regulates glucose metabolism by inhibiting α-glucosidase [31]. These findings suggest additional health benefits in the digestive tract, using the fruit’s rind in bakery foods. In addition, pomegranate pectin has found food-related applications as a gelling agent [32] and an emulsifier [33]. The properties and bioactive composition of pomegranate rind are what allows it to have several applications in skin health and food industries. Table 1 shows the chemical properties of the pomegranate seed and rind, which correspond to some of the potential uses of pomegranate biowaste in food additives.

In addition to fatty acids in PSO and the chemical properties of pomegranate seed and rind that make them suitable food additives, the phenolic content of pomegranate biowaste makes it a proper active ingredient for health and intelligent food packaging applications. Table 2 shows the total polyphenol, flavonoid, anthocyanin, and hydrolyzable tannin contents of pomegranate rind and seed that were obtained using aqueous and methanolic extraction methods. Methanolic extracts from pomegranate parts have been shown to have higher phenolic content than the aqueous extracts (Table 2).

Extractions. Extractions can be done using several different solvents. The yield of total weight equivalents to gallic acid for dry pomegranate rind peels using ultrasound-assisted extraction is the greatest for water, 50% aqueous methanol, ethanol, and then acetate [36]. Although water has a higher yield in extraction for antioxidant components in pomegranate, the extracts from water are not as effective as those of methanol, as methanol extracts show stronger antioxidant activities than water extracts through an ABTS radical scavenging assay [37,38]. However, water can be used to avoid using unhealthy organic solvents, like methanol, which has residues that compromise the extract’s use for food purposes by law. Therefore, additional research must be done on how to make water extractions more efficient or on finding other inexpensive, non-toxic solvents to the environment to gain both the health and efficacy benefits. Other extraction methods include non-conventional methods, such as ultrasound, microwave, and electrically pulsed fields, which have been shown to increase the efficiency of extraction and protect against oxidation and thermal degradation for bioactive components of pomegranate fruit, such as polyphenols [38]. 

One study showed the inhibiting effect of purified pomegranate rind as a source of polyphenol extract on the influenza virus, with punicalagin acting as the active ingredient [39]. Pomegranate rind is also a source of anthocyanins, a type of flavonoid that impacts the hue of food and food packaging, and it can potentially be used in intelligent packaging [40]. Table 2 shows that the pomegranate rind with methanolic extraction currently produces the highest phenolic content extract, which can then be used as active ingredients for various applications.

**Figure 4:**
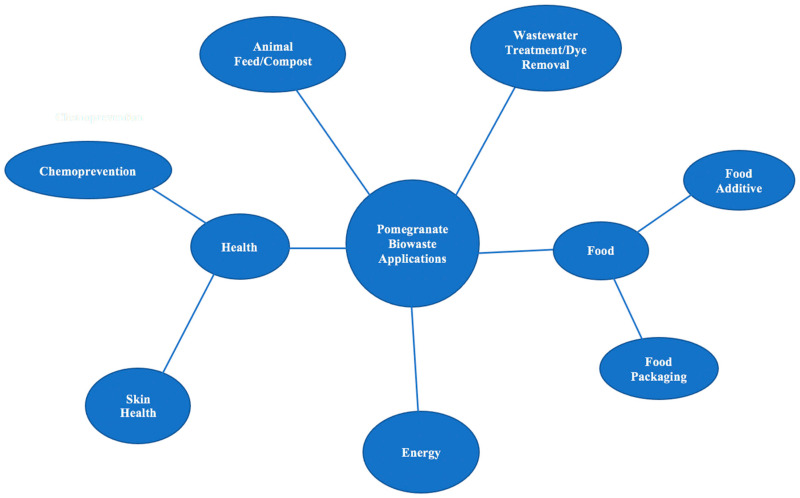
Applications of Pomegranate Biowaste.

This review focuses on the nutritional and bioactive components of pomegranate and the potential value that they have in food and skin health applications. This is an important topic to explore because, instead of using by-products of pomegranate juice for value-added applications, companies currently pay farmers to use pomegranate waste as animal feed. Although there are health benefits to converting pomegranate waste into animal feed [41,42,43,44,45], pomegranate waste has also been shown to be highly beneficial when incorporated into food and skin products. Figure 4 shows a graphical categorization of the known applications of pomegranate biowaste. Although there are many fields of research on pomegranates, this review targets those focusing on food and skin health value-added applications, with a focus on more recent findings.

## 2. Food Applications

Sustainability in the food and agriculture industry is compromised as enormous volumes of waste are generated; however, recycling and converting these wastes into value-added products for reuse in the food chain would revitalize sustainability. In this vein, this section will review the applications of pomegranate biowaste, like food additives and bioactive compounds for food packaging, in preventing food oxidation and the growth of pathogenic microorganisms. Pomegranate rind and seed act as sources of antioxidants, antimicrobial agents, and anti-browning agents for both food additives and food packaging material in various studies because of their many bioactive and nutritional components.

### 2.1. Food Additives

Nowadays, there is an increasing demand for natural alternatives to synthetic food ingredients and synthetic antioxidants, in particular, which have carcinogenic effects and have been restricted in use for food applications [46]. Pomegranate rind extract is an alternative natural antioxidant that is comparable to synthetic antioxidants, such as butylated hydroxytoluene (BHT). Pomegranate seed also has antimicrobial and antioxidant properties. In addition to acting as a substitute natural ingredient, pomegranate biowaste can replace commercial nutritional ingredients. For instance, pomegranate rind can serve as an appropriate pectin substitute, and PSP has the potential to be used as a source of nutritional fiber.

#### 2.1.1. Antioxidant and Antimicrobial

The “clean label” trend first appeared during the 1980s, and it referred to the idea that the food industry should be clear on whether certain ingredients or additives are present in food products or not, or if the food is produced by a more “natural” production method [47]. Companies have been reformulating their products with green alternatives to synthetic materials because of the increasing consumer demand for clean-label products.

Currently, in the food industry, synthetic antioxidants, such as BHT and butylated hydroxyanisole (BHA), are conventionally used to prevent oxidation. However, consumers generally prefer natural antioxidants, as they are deemed to be safer and healthier than synthetic antioxidants [48].

Lipid oxidation in meats negatively impacts their sensory properties (flavor, odor, texture, and color) and decreases their shelf life [49]. One study substituted synthetic antioxidants with pomegranate rind powder as a natural antioxidant in burger meats and then compared its effectiveness to that of BHT. The meat containing rind extract not only displayed higher sensory scores than the BHT-containing one, but it also displayed a lower aerobic bacterial count due to the presence of phenolic compounds in pomegranate rind [49]. In a similar study, pomegranate rind extract that was standardized to 40% ellagic acid was added to low-fat Kalari cheese as a preservative to improve the lipid oxidative stability. Cheese with pomegranate extract displayed higher sensory scores than the control sample, which may be attributed to the lower free fatty acid values, TBARS (assay to detect lipid oxidation) values, and a lowered microbial count due to the effect of bioactive components in the rind extract. Furthermore, significantly lower yeast and mold counts were observed after the addition of pomegranate rind extract to low-fat cheese [50]. 

With regard to the pomegranate seed’s antimicrobial properties, increasing the percentage of added pomegranate seed powder (PSP) decreased the standard plate and minimum yeast and mold counts in an ice cream formulation process [51]. In regard to pomegranate rind’s antioxidant properties, significant improvements in the antioxidant activity and α-glucosidase inhibition at rind phenolic levels of 0.5% *w*/*w* and 1.0% *w*/*w* were observed [52]. This trait is further emphasized by a new functional ingredient that is made of pomegranate rind combined with native corn starch in calcium alginate microspheres, which results in a higher free antioxidant capacity, similar to that of wheat bran [53]. 

Further studies support pomegranate rind acting in new functional ingredients and different carrier systems to improve the nutritional quality of food systems. For instance, the crude and encapsulated rind extract by stray drying can be enriched in hazelnut paste and other high lipid content food to increase the shelf life by inhibiting oxidation [36]. The extract has further applications as a functional ingredient with encapsulation by the orange juice industry by-products to increase antioxidant strength and replace synthetic antioxidants in the cosmetics and pharmaceutical industry [54]. From these studies, it is evident that the pomegranate rind and seed have chemical properties that help in both developing healthy food ingredients and enhancing a food product’s antioxidant and antimicrobial properties. In this way, pomegranate rind powder can be aptly compared to the synthetic antioxidant BHT while possessing health benefits. Pomegranate rind, in particular, is also a source of pectin, which has several commercial applications that will be discussed in the next section.

#### 2.1.2. Pectin

Commercial pectin has been used as a gelling, emulsifying, thickening, coloring, and stabilizing agent in the food industry. It can also replace fat or sugar in low-calorie foods [55]. Citrus fruits and apples are currently the main sources of commercial pectin with applications, such as protein stabilization in acidified dairy products [56]. However, pectin that was obtained from different sources can have varied effects on the product into which it is incorporated. Pectin composes 6.8–10.1% *w*/*w* of pomegranate rind, which is slightly lower than its content in commercial sources of pectin (apple pomace yields 10–15% *w*/*w* pectin and citrus rind yield 20–30% *w*/*w* pectin) [35]. Despite the existing lower pectin content of pomegranate rind, pomegranate rind pectin has been shown to still be an efficient agent, and it can even offer different results from commercial sources of pectin.

Pomegranate’s pectin was tested as a substitute gelling agent for commercial pectin in pomegranate jam. Overall, the sensory properties were acceptable as compared with the commercial pectin jam [32]. Although the commercial pectin jam and pomegranate rind pectin jam incorporated the same amount of anthocyanin pigments, the jam with pomegranate rind pectin was less red and yellow and darker than the control jam due to the different characteristics of the added pectin [32]. This experiment demonstrated the dual potential of pomegranate pectin as a natural gelling and coloring agent.

Moreover, proteins, polysaccharides, and some esters are commonly used as food emulsifiers. Pomegranate pectin is a polysaccharide that can be used as a natural emulsifier in the food industry. When pomegranate pectin was dissolved in water at different concentrations and then tested via homogenization as an emulsifier for coleseed oil, it was found that the emulsions were stable at a pectin concentration of 2.0% *w*/*v* and a pH between 2.0–4.0, although the emulsifying capacity was between pH values of 2.0–8.0 [33]. From these experiments, it can be seen that pomegranate pectin is an appropriate substitute for commercial pectin, whether it is used for gelling, coloring, or emulsifying functions.

#### 2.1.3. Fiber

Dietary fiber is found in fruits, vegetables, and whole grains. A high fiber diet helps to alleviate constipation, maintain healthy bowel movements, lower cholesterol levels, and control blood sugar levels [57]. Pomegranate seed is shown to have a dietary fiber content of 17.33–27.84% *w*/*w* when six varieties of pomegranates from Turkey were tested (Table 2) [34]. 

Pomegranate seed powder (PSP) is a form of dietary fiber that can be used as a food additive. PSP was used to replace a percentage of lean meat in the formulation of chicken nuggets in a study. The results showed that chicken nuggets had increased sensory attributes with a 3% *w*/*w* incorporation of PSP. There was also a significant increase in the crude fiber content of the nuggets with increasing levels of PSP. However, there was decreased emulsion stability as a result of decreased pH and water holding capacity and increased fat due to the abundance of fatty acids in pomegranate seeds [22]. In a similar study on bread making, pomegranate seed flour was used to replace up to 5% *w*/*w* of the wheat flour without drastically changing the sensory qualities of the bread. The resulting bread was labeled as a good source of fiber with lower production costs [21]. This is especially important because the bran and germ of wheat are taken out when wheat is milled, which causes a marked decrease in the dietary fiber content of the flour. When a portion of wheat flour was replaced with punicic acid-rich PSP, the bread’s dietary fiber, punicic acid, total polyphenol content, and radical scavenging activity were all increased. However, incorporating a 10% *w*/*w* level of PSP led to a slight decrease in the dough’s volume, crumb hardness, rheological properties, and sensory scores [58]. Pomegranate rind is also a source of dietary fiber and, when supplemented in cookies at an acceptable level of below 7.5% *w*/*w*, increases the crude fiber content by 80%. There was overall acceptability of the cookies, although this addition may have been attributed to the hardening of the cookies and a decline in sensory scores [30]. 

Because fiber is an important nutrient needed to maintain healthy stool and bowel movements, the above studies show the merits of pomegranate in adding fiber to food products, such as chicken nuggets and dough, without drastically changing their sensory scores or quality. They also show the potential of pomegranate to be used as a source of fiber in other food products, such as granola bars. Pomegrain bars have been formulated in one study in optimized conditions with 55% *w*/*w* PSP.

### 2.2. Food Packaging/Bioplastics

Table 2 shows the phenolic content of pomegranate rind and seed extracts. Several studies show the uses of the pomegranate biowaste’s phenolic content as active components in biodegradable and edible films and coatings. The following sections discuss pomegranate extract’s antioxidant and antimicrobial properties, coloring effects, plasticizing effects, and strengthening abilities for food packaging films. Pomegranate biowaste has promising impacts on sustainability for bioplastics because the biowaste can be used to fortify materials that can replace plastics in packaging systems.

#### 2.2.1. Active Ingredient with Antioxidant and Antimicrobial Effects

Pomegranate rind extract is able to enhance the antioxidant properties of films and coatings due to its high phenolic content (PC) [59]. More specifically, punicalagin, which is the predominant ellagitannin in pomegranate, is the phenolic component in pomegranate rind that is responsible for its antioxidant activity [60]. This is evident in k-carrageenan films, where the addition of non-edible pomegranate rind extract improved the UV light barrier and antioxidant properties of the films in comparison with edible pomegranate flesh extract. These findings can be attributed to the pomegranate rind’s relatively higher total PC [40]. Furthermore, in both k-carrageenan films and fish gelatin biopolymer packaging, the high PC of pomegranate rind improved the antioxidant activity during 2,2-Diphenyl-1-picrylhydrazyl (DPPH) radical scavenging tests to 59.74% at 1% *w*/*w* pomegranate rind and 71.82% at 5% *w*/*w* pomegranate rind. The antioxidant potential is due to pomegranate’s phenolic hydroxyls acting as reducing agents of free radicals [59,60]. There was also a direct positive correlation between the amount of pomegranate rind powder added to fish gelatin film and PC content in the film [60]. The same correlation was also observed between the total PC and antioxidant activity of biodegradable zein biopolymer film [61]. These observations further emphasize the pomegranate rind’s effectiveness as an antioxidant additive in bioplastics. 

The PC of pomegranate rind has also shown great potential as an antimicrobial component in bioplastics due to its ability to chelate vitamins, carbohydrates, and minerals, which makes them unavailable to bacteria [62]. Tannins (punicalagin) and polyphenols (ellagic acid) are the major components that contribute to this effect [63]. In one study, pomegranate rind extract that was encapsulated on chitosan nanoparticles was incorporated into biodegradable zein biopolymer film. The film showed the strong antimicrobial effect on L. monocytogenes bacteria during meat preservation. The antimicrobial activity of hydrolyzable polyphenols in the extract originates from the precipitation and lysing of bacterial cell membranes through the reaction of the rind phenolics [61]. While using the rind component of pomegranate for its antimicrobial effects in a study, the addition of ground pomegranate rind powder, a rich source of PC, to fish gelatin biopolymer packaging displayed strong antimicrobial effects, especially on gram-positive bacteria (e.g., s. Aureus and L. monocytogenes), while it weakly affected gram-negative bacteria (e.g., *E. coli*) [60]. The same results were obtained in studies where pomegranate rind powder was added to mung bean protein films [63] and starch-based films. This time, improved antimicrobial activity against Salmonella as a gram-negative bacteria was achieved [64]. 

Edible coatings made from chitosan and pomegranate rind extract have been shown to prevent microbiological food spoilage. As a coating, this combination lowered the total aerobic plate counts (the total amount of bacteria) and prevented the pH increase and the total volatile basic nitrogen (TVB-N) of pacific white shrimp within iced storage [65]. It also slowed the increase of the chemical spoilage values, such as peroxide value, TVB-N, and TBARS, for Nile tilapia fillets [66]. These studies reiterate the effectiveness of pomegranate rind as an antimicrobial agent.

#### 2.2.2. Packaging Color

Anthocyanins are the most prevalent flavonoids in pomegranates, and they are responsible for the orange, red, and purple colors of the fruit. They can act as effective additives in films due to their inherent properties as flavonoids by changing the color of the packaging material to reduce food oxidation. For example, the addition of pomegranate rind extract in chitosan-starch film reduces its transparency [62]. This would also significantly alter the color of edible films containing pomegranate extract by increasing the redness [63]. The decrease in transparency and increase in the redness of films caused by the anthocyanins help to inhibit the oxidation of packaged foods that result from their exposure to light [60]. Similarly, incorporating rind extract into chitosan coating for shrimp inhibited melanosis browning and improved sensory and texture qualities [65]. The addition of 1 *w*/*v* rind extract to chitosan delayed the ripening and browning process in guava fruit by slowing the respiration rate [67]. 

Pomegranate flesh extract has a relatively higher anthocyanin content than non-edible pomegranate rind extract. Consequently, when testing the effects of the extracts on the k-carrageenan film, pomegranate flesh extract displayed a higher sensitivity to pH-related color changes in the film than the rind extract. This suggests a possible application of anthocyanin-rich pomegranate flesh extract in examining the freshness of the packaged food by indicating the degree of oxidization in it [40]. However, anthocyanins can affect the visual appearance of food when added to edible coatings. For example, yellow-amber hues formed on shrimp in chitosan and locust bean gum coating mixed with pomegranate rind extract [68].

Similarly, the pomegranate’s anthocyanins show great potential as an active ingredient for intelligent packaging. Anthocyanins have the ability to slow oxidation and respiration rate and respond to pH changes of the system through color changes in packaging films. Several other components in pomegranate rind and seed have plasticizing, strengthening, and elongating effects on bioplastics, which further improves the abilities of pomegranate biowaste as an effective active ingredient in food packaging system applications.

#### 2.2.3. Plasticizing, Strengthening, and Elongation Effects

Several components of pomegranates, including pectin and polyphenols in rind extracts and PSO, increase the plasticizing effect, tensile strength (TS), and elongation at break (EAB) of bioplastic films. The polyphenols of pomegranate rind increase the thickness and strength of films due to interactions of their phenolic hydroxyl groups and the functional groups of the biopolymer film [63]. Additionally, pectin, fiber, and starch from the rind of pomegranates were shown to improve the plasticizing effect and flexibility of mung bean protein films [63], and pectin from rinds increased the TS of fish gelatin films [60]. However, the EAB decreased when pomegranate rind pectin was used as the main material in biodegradable films with montmorillonite as an additive [69]. Incorporating pomegranate rind extract into a chitosan gel and gelatin-based food coating increased its stability and viscosity against applied strain as more extract was added, suggesting the possibility of using pomegranate in edible fruit coatings [59]. 

PSO can also be used as an additive in films and coatings to promote desired changes in the material. In an edible film made of whey protein and k-carrageenan, the addition of PSO had a plasticizing effect and decreased the film’s TS [70]. For an edible coating made from Chlorella sp., PSO was added to promote the emulsifying and plasticizing effects and increase the shelf life of fruits [71]. Although PSO extracts have shown great plasticizing effects on the packaging material in these studies, other studies have shown that rind extract and the essential oil is used as additives decreased the flexibility, TS, and EAB of films [62,72]. This finding suggests that PSO alone is a better additive to packaging films than the combination of PSO with essential oils due to these differences in plasticizing effects.

## 3. Skin Health Applications

Pomegranate rind and bioactive seed compounds can be integrated into skin health products, demonstrating the potential that biowaste can be converted into value-added products. Ellagic acid and punicalagin are both bioactive compounds of pomegranate rind that promote skin health by inhibiting tyrosinase and initiating anti-inflammatory and anti-fungal effects [73,74,75]. PSO is rich in punicic acid, which gives it protective and anti-inflammatory characteristics to act against UV-induced radiation [76]. Furthermore, PSO can act as an inhibitor for aging-induced glycation, a process that negatively affects skin elasticity [77]. The following outlines the pomegranate extract’s promising pharmaceutical and cosmetic applications, such as the treatment of UV-induced hyperpigmentation, decreased skin elasticity, and skin wrinkling.

### 3.1. Skin Whitening

Pomegranate has one of the highest levels of ellagic acid (EA) among fruits and vegetables. EA is a phenolic component that is used to protect skin against oxidative stress [78]. EA is currently approved as a lightening ingredient for cosmetic formulations due to its ability to chelate copper ions that are present in tyrosinase enzymes, which are the main enzymes catalyzing the production of melanin [79]. 

EA that is found in pomegranate has advantageous treatment abilities for UVB-induced hyperpigmentation [76]. In one study, pomegranate rind extract containing 90% *w*/*w* EA was orally administered to UV irradiated guinea pigs to test its skin whitening effect. The extract taken orally had a comparable whitening effect to L-ascorbic acid (vitamin C), which is a known tyrosinase inhibitor on UV-induced pigmentation, and reduced the number of DOPA-positive melanocytes, whereas L-ascorbic acid did not [73]. Apart from its skin whitening effect, EA in pomegranate has more skin health applications that will be discussed in the next sections.

### 3.2. Skin Wrinkling and Skin Aging

Pomegranate extract also has an anti-aging effect against skin wrinkling and it can increase skin elasticity. PSO can improve the striae distensae skin condition, which is associated with a lack of skin elasticity. It was tested in an oil-in-water cream with Croton lechleri resin, which increased the thickness, hydration, and elasticity values of the dermis [80]. Another topical oil-in-water emulsion was formulated with pomegranate extract, donkey milk, and UV filters. In addition to an overall decrease in brown pigmentation, the emulsion had anti-aging effects on the skin, such as a decreased wrinkle count by 32.9%, decreased wrinkle length by 9.6%, and increased skin firmness and elasticity by 9.6%. This suggests that these effects are due to the synergistic potency of the ingredients of the formulation [81]. Pomegranate EA, in particular, has the ability to prevent UVB-induced thickening of the dermis, a process that can lead to skin wrinkling [82]. 

Glycation, which is also known as the Maillard reaction, is a process that creates advanced glycation end products (AGEs), which is partly induced by aging [77]. It is also a non-enzymatic, irreversible reaction between reducing sugars and proteins [83]. Skin glycation affects collagen in a way that results in the deterioration of skin elasticity. The anti-glycation property of a polysaccharide fraction from pomegranate extract was studied by evaluating the content of fructosamine, an early glycation product. The pomegranate extract acted as a glycation inhibitor due to both its free radical scavenging ability and its inhibition of fructosamine formation by modification of the amino or carbonyl groups in the Maillard reaction [77]. More recently, oral dosages of 100 mg/day pomegranate extract were given to post-menopausal healthy females. The results showed a decrease in glycative stress markers in those who had received the extract dosages [84]. Pomegranate extract was also found to be effective in firming skin after weight change or cosmetic surgery, as it increased the synthesis of glycosaminoglycans in the skin [85]. In another application, pomegranate extract from sterols with shale extract was used in a lip gloss formulation to treat rough, dry, or cracked lips and reduce the appearance of wrinkles [86]. In short, pomegranate extract can be considered to be beneficial for eliminating wrinkles that are induced by skin aging and damage from UV.

### 3.3. Burn and Wound Healing

In addition to its skin whitening and anti-aging effects, EA from pomegranate rind extract has a protective effect on sunburns at low doses (100 mg/day EA) [87]. Pomegranate extract with 40% *w*/*w* EA also has a healing effect on deep second-degree burn wounds in rats through the induction of collagen formation, which strengthens wounded tissue and speeds up the healing process [88]. The same extract can also enhance the healing process for incision wounds on rats by increasing collagen content and angiogenesis while decreasing polymorphonuclear leukocytes (PMN) infiltration, which causes tissue damage during inflammation [89]. Furthermore, EA and pomegranate rind extract positively contribute to increasing tensile strength in rat incision wounds. Although a high dose of EA alone can inhibit PMN infiltration, it cannot produce significant amounts of collagen. This is an indication of the synergistic effect of pomegranate extract with EA on healing wounds [90].

### 3.4. Anti-Inflammatory and Anti-Pain

Pomegranate rind extract was tested on ex-vivo porcine skin for its anti-inflammatory effects. The punicalagin permeated the skin, thus downregulating COX-2, an inflammatory enzyme [74]. A hydrogel containing pomegranate rind extract and zinc sulfate was formulated by the same research team as a topical treatment for Herpes simplex virus (HSV) infection. The hydrogel exhibited virucidal and anti-inflammatory effects, with punicalagin permeating regions of the skin that are susceptible to infection [91]. This has relevance in the growing need for novel clinical products to combat HSV. Another study using the rind extract’s punicalagin with zinc (II) ions established virucidal and therapeutic effects against HSV infections, such as the common cold sore, in order to further emphasize this potential application [92].

In addition to bioactive components of pomegranate rind, PSO, when topically applied, alleviates oxidative and inflammatory stress brought on the skin by UV irradiation. A topical hydrogel that was formulated with silibinin-loaded pomegranate oil-based nanocapsules had an anti-inflammatory effect on mice skin damaged by UVB induced radiation [93]. Furthermore, PSO nanoemulsions can provide photoprotection against UVB-induced damage of DNA in human keratinocyte HaCaT cells, which constitute most of the epidermis [94]. In another study on keratinocyte cells, pomegranate extract phenolics, including punicalagin, EA, and urolithin A, had protective effects against hydrogen peroxide-induced oxidative stress and cytotoxicity [95]. These studies demonstrate the existing potential of pomegranate extracts for use in sunscreen and cosmetics products. Furthermore, the topical application of PSO decreased skin tumor development and multiplicity, and it has shown applications as a chemo-preventive agent for skin cancer [17].

### 3.5. Anti-Bacterial and Anti-Fungal

Pomegranate extract can inhibit the growth of several dermatophyte fungi, such as Trichophyton rubrum, Trichophyton mentagrophytes, Microsporum canis, and Microsporum gypseum, all of which can cause dermatophytosis infection of nails, skin, and hair, with punicalagin as the active component that prevents dermatophytosis. Pomegranate extract can be used as a source of punicalagin for this purpose because pomegranate punicalagin accounts for most of the minimum inhibitory concentration that is found in pomegranate extract [75]. Furthermore, pomegranate rind powder, honey, and bee venom were successfully incorporated into a nanofiber wound coating for excision wounds in rats, which suggests the synergistic effect of honey and pomegranate rind when incorporated into the anti-bacterial activity of the coating on *E. coli* [96].

On another note, pomegranate rind has more recently been shown to have medicinal and antiviral applications via its bioactive components. An in vitro study using the rind extract has demonstrated great potential in its polyphenols impeding interactions between SARS-CoV-2 Spike glycoprotein and the human Angiotensin-Converting Enzyme 2 receptor, a prominent way to curb the SARS-CoV-2 disease [97]. Another recent study established the rind extract and its phenolics with n-butanol fraction’s efficacy in treating the adenovirus, a human disease that can provoke serious infections in immunosuppressed subjects and has no approved treatment against it [92,98].

Overall, pomegranate extract’s EA is able to effectively treat UV-induced hyperpigmentation and help in the healing of skin burns and wounds. The extract shows potential as an anti-bacterial coating capable of being joined with its healing effects to reinforce a product. PSO also has applications in preventing and productively decreasing skin wrinkling that is caused by aging and UV radiation. 

Commercial EA is procured by chemical extractions that hydrolyze rich-ellagitannin plants. Pomegranate fruit has a high polyphenol content, and it has been concluded in one study to have good by-product components for fermentation systems in order to retrieve high-value commercial compounds [99]. Hydrolyzed extracts of pomegranate rind were found to have a high EA content, at 12% of the dry weight of the fruit. Only about 9% of the total EA content is considered to be free ellagic acid in the rind extract. Therefore, most of the EA comes from hydrolysis of ellagitannins, and it is transformed from the phenolic compound gallic acid [100].

## 4. Summary

Many recent studies in the past year focused on the pomegranate’s synergistic effects with different bioplastic materials, particularly in food packaging. Pomegranate rind and seeds have strong antioxidant and antimicrobial properties that make them suitable natural alternatives to synthetic components in bioplastics, food coatings, and food additives. Pomegranate, as a natural source of pectin and fiber, has effects that are comparable to commercial pectin in terms of emulsifying and gelling properties while also providing health benefits from its increased fiber content as a food additive. 

There has also been a focus in recent studies on ellagic acid in pomegranate rind, especially in skin health applications for its protective effect against UV irradiation-induced stresses, such as hyperpigmentation, skin aging, sunburns, and skin cancer. Because many of these studies have tested animals, future research should focus on humans to better understand the full potential of pomegranate in food and skin health applications to promote broader commercial uses. It would be valuable to further explore the commercial uses of pomegranate in an economically and environmentally sustainable fashion by adding value to its waste. Moreover, there is a need for cost-effective techniques for the extraction of nutritional and bioactive components of pomegranate biowaste and for studies to be conducted on larger scales. Pomegranate rind and rind extract have an underlying astringency that restrains it from being used as an ingredient in food systems, so more investigation should be done to address this hurdle in food additive applications [101]. Finally, more research must be done on the interaction between food ingredients and the bioactive components of pomegranate. Taking these steps will not only give us a better understanding of pomegranate waste utilization methods, but will also advance research in the fields of skin health and food science.

## Figures and Tables

**Table 1:** Chemical properties of pomegranate seed and rind by dry weight [34,35].

Property	Seed Amount	Rind Amount
Moisture	10.44–12.86%	67.26–73.23%
Sugar	N/A	30.65–34.83%
Crude Oil	10.89–13.24%	N/A
Crude Protein	6.71–8.11%	3.96–7.13%
Crude Ash	1.61–2.29%	3.71–4.97%
Fiber	17.33–27.84%	28.10–33.93%
Pectin	N/A	6.8–10.1%

**Table 2:** Total polyphenol, flavonoid, anthocyanin, and hydrolyzable tannins in pomegranate rind and seed [37].

	Total Polyphenol (mg/g * GAE **)		Total Flavonoid (mg/g RE ***)		Total Anthocyanin (mg/g CGE ****)		Hydrolyzable Tannins (mg/g TAE *****)	
Extraction Method	Aq.	MeOH	Aq.	MeOH	Aq.	MeOH	Aq.	MeOH
Rind	53.65 ± 4.13	85.60 ± 4.87	21.03 ± 1.62	51.52 ± 8.14	51.02 ± 10.33	102.20 ± 16.42	62.71 ± 11.32	139.63 ± 4.25
Seed	7.94 ± 1.25	11.84 ± 1.92	3.30 ± 0.52	6.79 ± 0.57	19.62 ± 3.12	40.84 ± 7.77	32.86 ± 4.24	29.57 ± 4.54

* mg/g of pomegranate seed, leaf, flower, and peel. ** GAE: Gallic Acid Equivalents. *** RE: Rutin Equivalents. **** CGE: Cyanidin-3-glucoside Equivalents. ***** TAE: Tannic Acid Equivalents.

## Data Availability

Not applicable.
